# The development of Friedländer heteroannulation through a single electron transfer and energy transfer pathway using methylene blue (MB^+^)

**DOI:** 10.1038/s41598-022-11349-8

**Published:** 2022-05-04

**Authors:** Farzaneh Mohamadpour

**Affiliations:** School of Engineering, Apadana Institute of Higher Education, Shiraz, Iran

**Keywords:** Chemistry, Photochemistry, Photocatalysis

## Abstract

The radical Friedländer hetero-annulation of 2-aminoaryl ketone and -methylene carbonyl compound was used to develop a green tandem approach for the metal-free synthesis of polysubstitutedquinolines. At room temperature in an ethanol solvent, photo-excited state functions generated from MB^+^ were used as single-electron transfer (SET) and energy transfer (EnT) catalysts, utilizing visible light as a renewable energy source in the air atmosphere. The purpose of this research is to increase the use of a nonmetal cationic dye that is both inexpensive and widely available. High yields, energy-effectiveness, high atom economy, time-saving features of the reaction, and operational simplicity, and the least amount of a catalyst are the benefits of this study. As a result, a wide range of ecological and long-term chemical properties are obtained. Polysubstitutedquinolines' turnover number (TON) and turnover frequency (TOF) have been calculated. Surprisingly, such cyclization can be accomplished on a gram scale, indicating that the process has industrial potential.

## Introduction

The use of photo-redox catalysts in organic synthesis for the formation of C–C and C–heteroatom bonds via a single-electron transfer (SET)/photo-induced electron transfer (PET) pathway has increased dramatically in recent years. They are essential in a wide range of procedures, from small to large-scale. Various flow reactors^1^ utilizing visible light and dual photosensitized electrochemical processes^2^ have been created as a result of technological advancements, resulting in more affordable, green, and efficient reactions. MB^+^ is a cationic dye in the thiazine dye class. MB^+^ has a singlet lifetime of τ_f_ ~ 1.0 ns, as well as an absorbance of near 650–670 nm (668 nm) and a molar absorbance (ε = 94,000)^3,4^. The triplet ^3^MB^+*^ is a significantly more stable excited state^5^, with a triplet lifespan of τ_f_ ~ 32 μs^5,6^. (More content and discussions about photoredox cycle catalyzed by dye^7^ have been added to the supporting information file).

Furthermore, because visible light irradiation has enormous energy reserves, lower prices, and renewable energy sources, green chemists consider it a dependable method for environmentally friendly organic chemical synthesis^8–10^. As visible light sources, compact fluorescent bulbs and light-emitting diodes are commonly used in many conversions.

**Figure 1:**
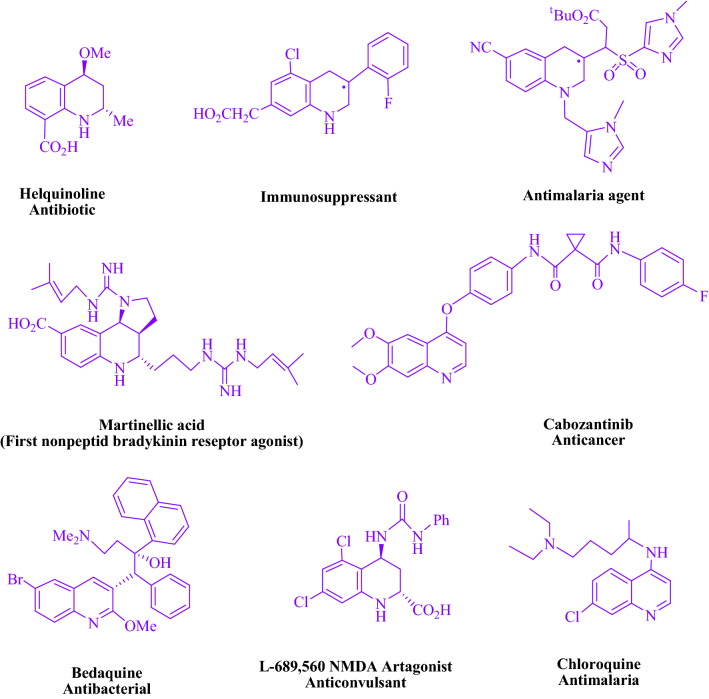
Compounds with biologically active quinolines rings.

The structures that make up quinolines have piqued the interest of biochemists and synthetic organic chemists due to their biological and pharmacological actions (Fig. 1). Quinolines have been described in the scientific literature as inhibit acetylcholinesterase^11^, butyrylcholinesterase family of enzymes^12^, antifilarial^13^, antiparasitic^14^, tyrosine kinase inhibitory agents^15^, HMG-CoA reductase inhibiting^16^, antitubercular^17^, antifungals^18,19^, antihypertensive^20,21^, antiallergic, antiinflammatory^22–24^, antibacterial^25–28^, antimalarials^29^, anticancer^30–33^, antiproliferative^34^ and antiasthmatic^35,36^. Quinoline nucleus can also be found in a variety of natural products^37–39^.

Numerous strategies are available, including DSIMHS^40^, Zn(OTf)_2_^41^, NiO NPs^42^, Zr(NO_3_)_4_^43^, I_2_^44^, PEG-bound sulfonic acid^45^, triflouroacetic acid^46^, propylsulfonic silica^47^, HClO_4_·SiO_2_^48^, Chitosan-SO_3_H^49^, oxalic acid^50^, Ag_3_PW_12_O_4_^51^, ImBuSO_3_H^52^, MNP@PEG-ImHSO_4_^53^. Metal catalyst limitations, expensive reagents, harsh reaction conditions, monotonous unacceptable yields, environmental risks, workup processes, and long reaction times have all resulted from these methods. Furthermore, it is difficult to separate a homogeneous catalyst from the reaction mixture.

We've been attracted by the hunt for easy, efficient, and environmentally acceptable techniques to synthesizing biologically active chemicals utilizing photocatalysts^54–56^ because of the aforementioned problems and our concern for environmentally favorable operations. Given prior and ongoing attempts to manufacture polysubstitutedquinolines, it's critical to investigate environmentally friendly photocatalysts in green environments to ensure that these heterocyclic compounds are properly synthesized. This research focuses on the utilization of MB^+^, a metal-free cationic dye photo-redox catalyst, in the aforementioned photochemical synthesizing technique. Finally, a green tandem strategy for the metal-free synthesis of polysubstitutedquinolines was developed using the radical Friedländer hetero-annulation^57^ of 2-aminoaryl ketone and -methylene carbonyl molecule. Photo-excited state functions produced from MB^+^ as single-electron transfer (SET) and energy transfer (EnT) catalysts were employed at room temperature in an ethanol solvent, exploiting visible light as a renewable energy source in the air atmosphere. The goal of this study is to increase the usage of an inexpensive and widely available nonmetal cationic dye. The benefits of this study include excellent yields, energy efficiency, high atom economy, time-saving aspects of the reaction, operational simplicity, and the use of the least amount of a catalyst. Furthermore, the use of organic solvents under reflux conditions, as well as the need for column chromatography to purify the products, is a source of environmental pollution. The products were produced with simple filtration and recrystallization with ethanol in this study, with no need for column chromatographic separation. Surprisingly, gram-scale cyclization is possible, indicating that the technique has industrial potential. This is a successful one-pot reaction that was carried out in a very efficient, cost-effective, and simple manner.

## Experimental

### General

All substances' physical properties are determined using electrothermal 9100 equipment. On a Bruker (DRX-300) device, the spectra (^1^HNMR) were also recorded using nuclear magnetic resonance with CDCl_3_ as the solvent. We purchased the reagents in bulk from the chemical companies Fluka, Merck, and Acros and used them exactly as they were.

#### General procedure for preparation of polysubstituted quinolines (3a-r)

MB^+^ (1 mol%) was added to a mixture of 2-aminoaryl ketone (**1**, 1.0 mmol) and -methylene carbonyl compound (**2**, 1.5 mmol) in EtOH (3 mL) and stirred at room temperature under white LED (12 W) irradiation. TLC was used to monitor the reaction's progress, with *n*-hexane/ethyl acetate as the eluent (3:2). Following the reaction, the resulting material was screened and washed with water, and the crude solid was crystallized again from ethanol to produce the pure substance without further purification. Even if we could produce the aforementioned compounds using gram scale methods, we wanted to see if we could scale up to the level required for pharmaceutical process R&D. In one experiment, 50 mmol 2-aminobenzophenone was mixed with 75 mmol acetylacetone. The large-scale reaction went off without a hitch and finished in just 6 min, with the product collected using simple filtration, rinse with water and then recrystallize with ethanol. This material's ^1^HNMR spectrum indicates that it is spectroscopically pure.

After comparing spectroscopic data, the commodities were classified. After comparing spectroscopic data, the commodities were classified (^1^HNMR).

#### 1-(2-Methyl-4-phenylquinolin-3-yl)ethanone (3k)


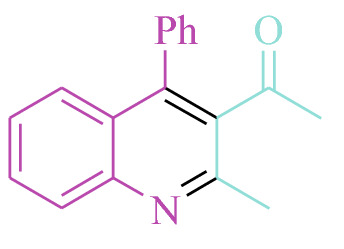


Yield: 94%; M.p. 110–112 °C; ^1^HNMR (300 MHz, CDCl_3_): 2.03 (3H, s, CH_3_), 2.65 (3H, s, CH_3_), 7.39–7.46 (6H, m, ArH), 7.53 (1H, d, *J* = 7.2 Hz, ArH), 7.64–7.66 (1H, t, *J* = 7.2 Hz, ArH), 8.02 (1H, d, *J* = 8.4 Hz, ArH).

#### 1-(6-Chloro-2-methyl-4-phenylquinolin-3-yl)ethanone (3l)


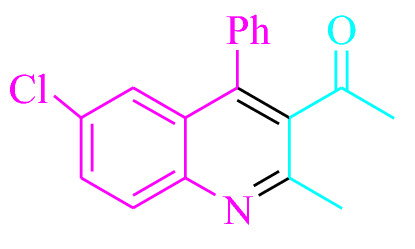


Yield: 97%; M.p. 152–154 °C; ^1^HNMR (300 MHz, CDCl_3_): 2.01 (3H, s, CH_3_), 2.69 (3H, s, CH_3_), 7.36–7.41 (2H, m, ArH), 7.50–7.59 (5H, m, ArH), 8.04 (1H, d, *J* = 8.4 Hz, ArH).

## Results and discussion

**Figure 2:**
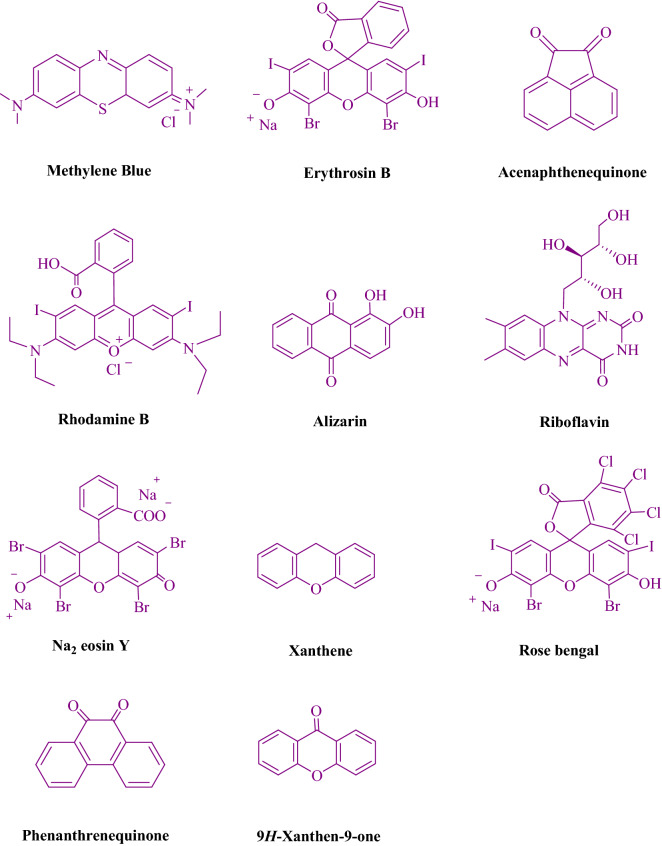
In this study, photocatalysts were put to the test.

**Figure 3:**
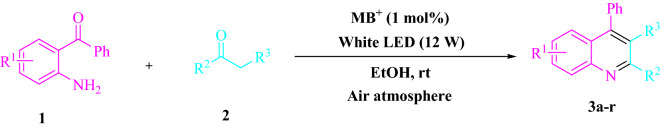
Polysubstitutedquinoline synthesis.

To begin, the reaction of 2-aminobenzophenone (1.0 mmol) and dimedone (1.5 mmol) in EtOH (3 mL) at room temperature was studied under LED irradiation. There was a trace of **3a** at rt in 3 mL EtOH for 40 min with no photocatalysts (Table 1, entry 1). Methylene blue, erythrosin B, acenaphthenequinone, rhodamine B, alizarin, riboflavin, Na_2_ eosin Y, xanthene, rose Bengal, phenanthrenequinone, 9*H*-xanthen-9-one (Fig. 2) were all tested in identical conditions to promote the reaction. This reaction progressed in 55–94% yields while achieving the acceptable matched product **3a** (Table 1). According to the findings, methylene blue fared better in such a response. The yield was increased to 94% by using 1 mol% MB^+^ (Table 1, entry 4). THF, toluene, DMSO and DMF all had lower product yields, as shown in Table 2. In H_2_O, H_2_O/EtOH (1:1), MeOH, EtOAc, CH_3_CN, and solvent-free conditions, the reaction rate and yield were increased. The reaction was carried out in EtOH at an excellent yield and rate. Under identical conditions, a yield of 94% was obtained, as shown in Table 2 (entry 2). Different light sources were used to screen the yield, demonstrating the effect of white light (Table 2). There was a minuscule of **3a** without using the light source, according to the test control. According to the findings, visible light and MB^+^ are required for the successful synthesis of product **3a**. Furthermore, the improved settings were determined by illuminating white LEDs of varying intensities (10, 12, and 18 W). The best results, according to the researchers, were obtained when white LED (12 W) were used (Table 2, entry 2). A wide range of substrates were investigated under the right conditions (Table 3 and Fig. 3). It is worth noting that the methylene carbonyl compounds had no effect on the reaction's outcome (Table 3). The reaction patterns of 2-aminobenzophenone and 5-chloro-2-aminobenzophenone were comparable (Table 3). Table 4 also includes turnover number (TON) and frequency of turnover information (TOF). The greater the TON and TOF numerical values, the less catalyst is used and the greater the yield, and the catalyst becomes more effective as the value increases. ^1^HNMR data some of known products has also been compared to literature (Table S1). (In the supporting information file, Table S1 has been added.)

**Table 1:** Table of photocatalyst optimization for **3a** production.


Entry	Photocatalyst	Solvent (3 mL)	Time (min)	Isolated yields (%)
1	–	EtOH	40	Trace
2	Methylene blue (0.2 mol%)	EtOH	20	56
3	Methylene blue (0.5 mol%)	EtOH	10	77
**4**	**Methylene blue (1 mol%)**	EtOH	**7**	**94**
5	Methylene blue (1.5 mol%)	EtOH	7	94
6	Erythrosin B (1 mol%)	EtOH	7	73
7	Acenaphthenequinone (1 mol%)	EtOH	7	56
8	Rhodamine B (1 mol%)	EtOH	7	78
9	Alizarin (1 mol%)	EtOH	7	55
10	Riboflavin (1 mol%)	EtOH	7	75
11	Na_2_ eosin Y (1 mol%)	EtOH	7	86
12	Xanthene (1 mol%)	EtOH	7	65
13	Rose bengal (1 mol%)	EtOH	7	70
14	Phenanthrenequinone (1 mol%)	EtOH	7	62
15	9*H*-Xanthen-9-one (1 mol%)	EtOH	7	58

Reaction conditions: At room temperature, 2-aminobenzophenone (1.0 mmol) and dimedone (1.5 mmol) in EtOH were used, along with a white LED (12 W) and a variety of photocatalysts.

Significant values are in bold.

**Table 2:** Table of solvent and visible light optimization for **3a** synthesis.


Entry	Light source	Solvent (3 mL)	Time (min)	Isolated yields (%)
1	White light (12 W)	H_2_O	7	85
**2**	**White light (12 W)**	**EtOH**	**7**	**94**
3	White light (12 W)	H_2_O/EtOH (1:1)	7	89
4	White light (12 W)	MeOH	9	82
5	White light (12 W)	EtOAc	10	51
6	White light (12 W)	CH_3_CN	7	80
7	White light (12 W)	–	20	57
8	White light (12 W)	THF	30	33
9	White light (12 W)	Toluene	30	27
10	White light (12 W)	DMSO	35	24
11	White light (12 W)	DMF	30	38
12	White light (10 W)	EtOH	7	83
13	White light (18 W)	EtOH	7	94
14	–	EtOH	35	Trace
15	Blue light (12 W)	EtOH	7	87
16	Green light (12 W)	EtOH	7	81

Reaction conditions: 2-aminobenzophenone (1.0 mmol) and dimedone (1.5 mmol) were added to MB^+^ at room temperature (1 mol%).

Significant values are in bold.

**Table 3:** Using photoexcited MB^+^ as a catalyst, this photocatalyst produces polysubstitutedquinolines.


**3a** (7 min, 94%) Mp. 194–196 °C Lit. 192–194 °C^40^	**3b** (7 min, 92%) Mp. 206–208 °C Lit. 207–209 °C^40^	**3c** (5 min, 97%) Mp. 137–139 °C Lit. 139–141 °C^46^	**3d** (5 min, 96%) Mp. 161–163 °C Lit. 162–164 °C^46^
**3e** (5 min, 93%) Mp. 196–198 °C Lit. 195–197 °C^53^	**3f.** (7 min, 96%) Mp. 199–201 °C Lit. 200–202 °C^53^	**3 g** (7 min, 95%) Mp. 97–99 °C Lit. 98–100 °C^52^	**3 h** (7 min, 93%) Mp. 106–108 °C Lit. 105–106 °C^52^
**3i** (7 min, 96%) Mp. 154–156 °C Lit. 156–158 °C^46^	**3j** (7 min, 97%) Mp. 187–189 °C Lit. 185–189 °C^46^	**3 k** (6 min, 94%) Mp. 110–112 °C Lit. 113 °C^42^	**3 l** (7 min, 97%) Mp. 152–154 °C Lit. 152 °C^42^
**3 m** (9 min, 92%) Mp. 216–218 °C Lit. 218–219 °C^52^	**3n** (10 min, 90%) Mp. 239–241 °C Lit. 238–239 °C^52^	**3o** (6 min, 93%) Mp. 105–107 °C Lit. 106 °C^42^	**3p** (6 min, 94%) Mp. 137–139 °C Lit. 136 °C^42^
**3q** (5 min, 95%) Mp. 128–130 °C Lit. 131–132 °C^46^	3r (5 min, 93%) Mp. 102–104 °C Lit. 104–106 °C^46^

**Table 4:** Calculated turnover number (TON) and turnover frequency (TOF).

Entry	Product	TON	TOF	Entry	Product	TON	TOF
1	**3a**	94	13.4	10	**3j**	97	13.8
2	**3b**	92	13.1	11	**3k**	94	15.6
3	**3c**	97	19.4	12	**3l**	97	13.8
4	**3d**	96	19.2	13	**3m**	92	10.2
5	**3e**	93	18.6	14	**3n**	90	9
6	**3f**	96	13.7	15	**3o**	93	15.5
7	**3g**	95	13.5	16	**3p**	94	15.6
8	**3h**	93	13.2	17	**3q**	95	19
9	**3i**	96	13.7	18	**3r**	93	18.6

**Figure 4:**
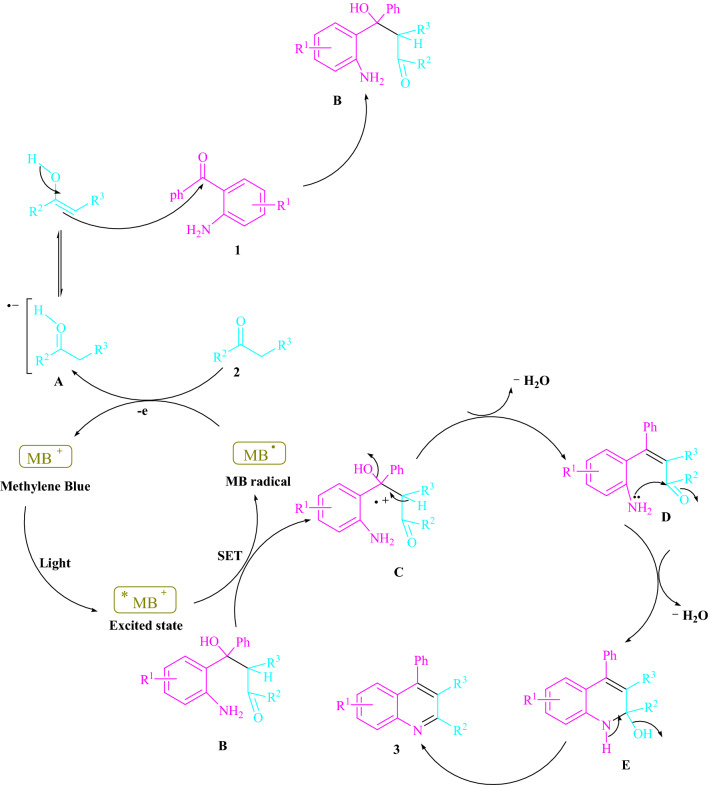
A mechanistic method for producing polysubstitutedquinolines has been proposed.

Figure 4 denotes the preferred mechanism. Photoexcited modes derived from methylene blue can act as a single-electron transfer (SET) and energy transfer (EnT) catalyst. The ground-state MB and the intermediate (**A**) are regenerated by an electron transfer (ET) between the MB radical and the -methylene carbonyl compound (**2**). A reactive intermediate (**B**) is formed when this radical anion (**A**) is nucleophilically added to 2-aminoaryl ketone (**1**). A single-electron transfer (SET) mechanism promotes the production of the cation radical (**C**) by visible light-triggered ^*^MB^+^. The dehydrated cyclized is then added for a total of **3**.

Table 5 compares the catalytic capability of various catalysts discussed in this literature for the synthesis of polysubstitutedquinolines. It could have a variety of applications, including the use of a small amount of photocatalyst, a fast reaction time, and the absence of by-products when exposed to visible light. The atom-economic protocol is extremely successful at multigram scales and has significant industrial implications. These materials stand out in terms of efficiency and purity.

**Table 5:** Comparison of the catalytic ability of some of the catalysts in the manuscript to produce **3a**, **3b.**

Entry	Compound	Catalyst	Conditions	Time/yield (%)	References
1		DSIMHS	Solvent-free,70 °C	25 min/89	^40^
2		Zn(OTf)_2_	Solvent-free, MW	5 min/86	^41^
3		NiO NPs	EtOH, Reflux	2.5 h/94	^42^
4		Zr(NO_3_)_4_	H_2_O, Reflux	30 min/98	^43^
5		Triflouroacetic acid	Solvent-free,100 °C	15 min/92	^46^
**6**		**MB**^**+**^	**Visible light irradiation, EtOH, rt**	**7 min/94**	**This research**
7		DSIMHS	Solvent-free,70 °C	40 min/90	^40^
8		Zn(OTf)_2_	Solvent-free, MW	5 min/88	^41^
9		NiO NPs	EtOH, Reflux	2 h/93	^42^
10		Zr(NO_3_)_4_	H_2_O, Reflux	25 min/98	^43^
11		Triflouroacetic acid	Solvent-free,100 °C	15 min/95	^46^
**12**		**MB**^**+**^	**Visible light irradiation, EtOH, rt**	**7 min/92**	**This research**

Reaction conditions: 2-aminobenzophenone/5-chloro-2-aminobenzophenone and dimedone.

## Conclusion

The photo-excited state functions generated by MB^+^ can be used to metal-free manufacture polysubstitutedquinolines via radical Friedländer hetero-annulation of 2-aminoaryl ketone and -methylene carbonyl compound via a single-electron transfer (SET)/energy transfer (EnT) method, according to the findings. This procedure employs visible light as a renewable energy source in an EtOH solvent and air atmosphere at room temperature. The use of the least amount of catalyst, excellent yields, an efficient side of the reaction, secure reaction conditions, a renewable energy source, and a quick procedure without the use of toxic solvents or catalysts are the most noticeable features of this green protocol. No chromatographic purification was required. According to a multigram scale reaction of model substrates, this reaction can be scaled up without compromising the outcome. As a result, this process provides additional benefits in terms of meeting industrial requirements and addressing environmental concerns.

## Supplementary Information

Supplementary Information.

## Data Availability

All data generated or analyzed during this study are included in this published article [and its supplementary information files].
