# Supplementary material for: The development of Friedländer heteroannulation through a single electron transfer and energy transfer pathway using methylene blue (MB+)
Source: Sci Rep. 2022 May 4;12:7253. doi: 10.1038/s41598-022-11349-8 (PMC9068815; doi:10.1038/s41598-022-11349-8)
Supplement: Supplementary file 1 — Supplementary Information. [file 41598_2022_11349_MOESM1_ESM.docx]

**Supporting information**

**The development of Friedländer heteroannulation through a single electron transfer and energy transfer pathway using methylene blue (MB^+^)**

Farzaneh Mohamadpour ^*^

School of Engineering, Apadana Institute of Higher Education, Shiraz, Iran

* Corresponding author. [mohamadpour.f.7@gmail.com](mailto:mohamadpour.f.7@gmail.com)

**Table of content**

1. Photoredox cycle catalyzed by dye.
2. Tables
   1. Table S1. Comparison of ^1^HNMR data.
3. References
4. **Photoredox cycle catalyzed by dye**

The photoredox cycle is started when dye in the ground state is irradiated with visible light to produce the high-energy excited state of dye (Dye^*^). The process of visible light photoredox catalysis is presented using two separate paths from dye in the excited state (Dye^*^). In the presence of a sacrificial electron acceptor, Dye* reductive's property can be employed. In other words, Dye^*^ leads the radical cation species of Dye as an electron donor. In the presence of a sacrificial electron donor, Dye^*^ also works as an electron acceptor [7].

The two mechanistic methods are used in most photoredox catalytic processes [3, 5]. The primary direction of electron transfer (ET) with respect to the excited state catalyst is classified in each of these photo-induced electron transfer (PET) cycles: in an oxidative quenching cycle, the excited state catalyst is quenched by donating an electron to substrate or an oxidant present in the reaction mixture; in a reductive quenching cycle, catalyst^*^ is quenched by accepting an electron from substrate or a reductant. In the oxidative cycle, reduction of the oxidized [catalyst]^•+^ occurs, while in the reductive cycle, oxidation of the reduced [catalyst]^•^ occurs. Catalyst turnover could be caused by the substrate, an external redox-active reagent, or an intermediary in either instance. There are three main redox outcomes for the substrate in either quenching manifold: net oxidative, net reductive, and net redox-neutral, regardless of whether the substrate performs an electron transfer (ET) reaction in the photo-induced electron transfer (PET) step or the turnover step. An external oxidant is required for a net oxidative reaction, which can take electrons in either the PET or turnover steps. Similarly, during the PET or turnover phases of net reductive processes, an external reductant donates electrons. Return electron transfer with the oxidized or reduced catalyst, occasionally mediated by a redoxactive co-catalyst, is common in net redox-neutral reactions [3].

1. **Tables**

| **Table S1.** Comparison of ^1^HNMR data. | | | | |
| --- | --- | --- | --- | --- |
| Entry | Product | H Shift (Found) | H Shift (Lit.) | Reference |
| 1 | **3k** | 2.03 (3H, s, CH_3_)  2.65 (3H, s, CH_3_)  7.39–7.46 (6H, m, ArH)  7.53 (1H, d, *J* = 7.2 Hz, ArH)  7.64–7.66 (1H, t, *J*= 7.2 Hz, ArH) 8.02 (1H, d, *J* = 8.4 Hz, ArH) | 1.96 (3H, s)  2.68 (3H, s)  7.35–7.50 (6H, m)  7.60 (1H, d, *J* = 7.9 Hz)  7.68–7.70 (1H, t, *J* = 7.9 Hz)  8.05 (1H, d, *J* = 8.0 Hz) | [51] |
| 2 | **3l** | 2.01 (3H, s, CH_3_)  2.69 (3H, s, CH_3_)  7.36–7.41 (2H, m, ArH)  7.50–7.59 (5H, m, ArH)  8.04 (1H, d, *J* = 8.4 Hz, ArH) | 2.00 (3H, s)  2.68 (3H, s)  7.33–7.35 (2H, m)  7.53–7.66 (5H, m)  8.00 (1H, d, *J* = 8.9 Hz) | [44] |

1. **References**

[3] Romero NA, Nicewicz DA. Organic photoredox catalysis. Chemical reviews. 2016; 116:10075-166.

[5] Patel RI, Sharma A, Sharma S, Sharma A. Visible light-mediated applications of methylene blue in organic synthesis. Organic Chemistry Frontiers. 2021; 8: 1694-718.

[7] Miyabe H. Organic Reactions Promoted by Metal-Free Organic Dyes Under Visible Light Irradiation. In Visible-Light Photocatalysis of Carbon-Based Materials. 2017; IntechOpen.

[44] Wu J, Xia HG, Gao K. Molecular iodine: a highly efficient catalyst in the synthesis of quinolines via Friedländer annulation. Organic & Biomolecular Chemistry. 2006; 4: 126-9.

[51] Yadav JS, Reddy BS, Sreedhar P, Rao RS, Nagaiah K. Silver phosphotungstate: A novel and recyclable heteropoly acid for Friedländer quinoline synthesis. Synthesis. 2004; 2004: 2381-5.
